# Crystal structure of (−)-(*S*)-4-[(2*S*,3*S*,4*S*,*Z*)-3-hydroxy-4-methyl­hept-5-en-2-yl]-1,3-dioxolan-2-one

**DOI:** 10.1107/S2056989017009318

**Published:** 2017-06-27

**Authors:** Keyla F. Morales-Rivera, Dalice M. Piñero Cruz, Jose A. Prieto

**Affiliations:** a Department of Chemistry, University of Puerto Rico-Rio Piedras Campus, PO Box 23346, San Juan, 00931-3346, Puerto Rico

**Keywords:** crystal structure, polypropionate, 1,2-carbonate, stereo­tetra­ds, O—H⋯O hydrogen bonding

## Abstract

The title compound, C_11_H_18_O_4_, consists of an *anti*,*anti*,*anti*-stereo­tetrad with a 1,2-carbonate and an alkene motif. The mol­ecule displays a common zigzag conformation. The five-membered ring has a twisted conformation on the C—C bond. In the crystal, a strong inter­molecular hydrogen bond between the hy­droxy group and the carboxyl­ate moiety from an adjacent mol­ecule forms chains propagating along the *b*-axis direction. The absolute structure of the mol­ecule in the crystal was determined by resonant scattering [Flack parameter = 0.05 (6)].

## Abstract 2

The title compound consists of an *anti*,*anti*,*anti*-stereo­tetrad with a 1,2-carbonate and an alkene motif.

## Chemical context   

The title compound was obtained as part of our studies toward the synthesis of (−)-dolabriferol and (−)-dolabriferol B (Ciavatta *et al.*, 1996 ▸; Jiménez-Romero *et al.*, 2012 ▸), using an epoxide-based approach for the stereo­tetrad construction. Polypropionate chains are structural motifs consisting of alternating methyl and hy­droxy groups within an aliphatic framework (Torres *et al.*, 2004 ▸, 2009 ▸; Tirado *et al.*, 2005 ▸, Rodríguez *et al.*, 2006 ▸). Their structure is found in various natural products, many of them possessing a wide range of biological activity, typically anti­biotic, anti­tumor, anti­fungal, anti­parasitic, among others (Rohr, 2000 ▸). Different method­ologies for the synthesis of polypropionates have been developed, with aldol and aldol-related chemistry being the most used (Schetter & Mahrwald, 2006 ▸).
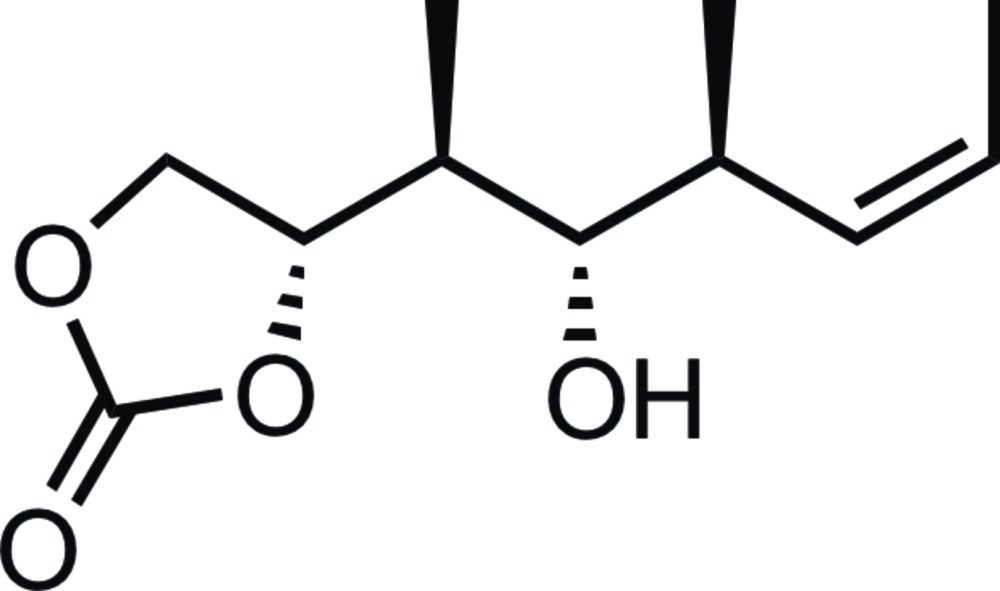


In our laboratory, we have developed an epoxide-based methodology for the construction of polypropionates, consisting of a reiterative sequence of three steps. Our approach involves a regioselect­ive epoxide cleavage with an alkynyl aluminium reagent (Torres *et al.*, 2005 ▸) or Grignard reagent (Rodríguez *et al.*, 2006 ▸), *cis* or *trans* reduction of the alkyne (if needed), and the stereoselective epoxidation of the resulting alkenol for the elaboration of each propionate unit. In this approach, the configuration of the hydroxyl group is derived from the absolute configuration of the epoxide precursor, while the *syn/anti* relative configuration of the methyl and hydroxyl groups is derived from the epoxide geometry. One of the advantages of this methodology is that it is a substrate-controlled synthesis; the only enanti­omeric step in this sequence is the first epoxidation (Katsuki & Sharpless, 1980 ▸).

## Structural commentary   

**Figure 1:**
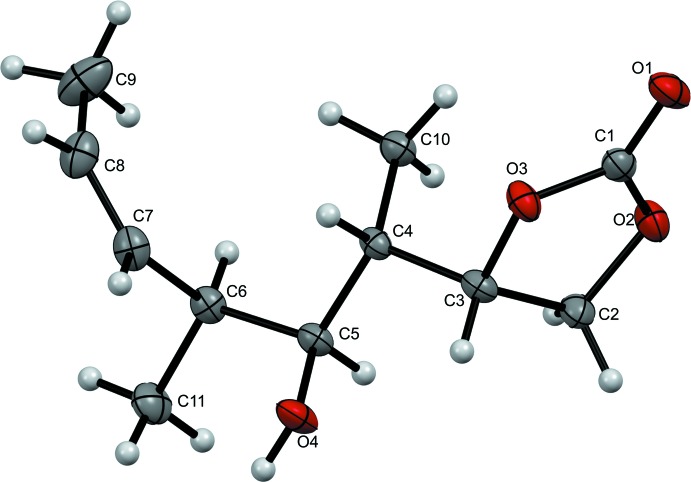
The mol­ecular structure of the title compound, with the atom labelling. Displacement ellipsoids are drawn at the 50% probability level.

The mol­ecular structure of the title compound is illustrated in Fig. 1 ▸. The alkyl back bone has a typical zigzag conformation with two of the three methyl groups, those located on C4 and C6, *anti* to one another. Likewise, the hy­droxy group located on C5 is in an *anti* relative conformation with respect to the methyl groups. The five-membered ring (O2/O3/C1–C3) has a twisted conformation on bond C2–C3 [puckering parameters *Q*(2) = 0.137 (2) Å and φ(2) = 307.4 (10)°].

## Supra­molecular features   

**Figure 2:**
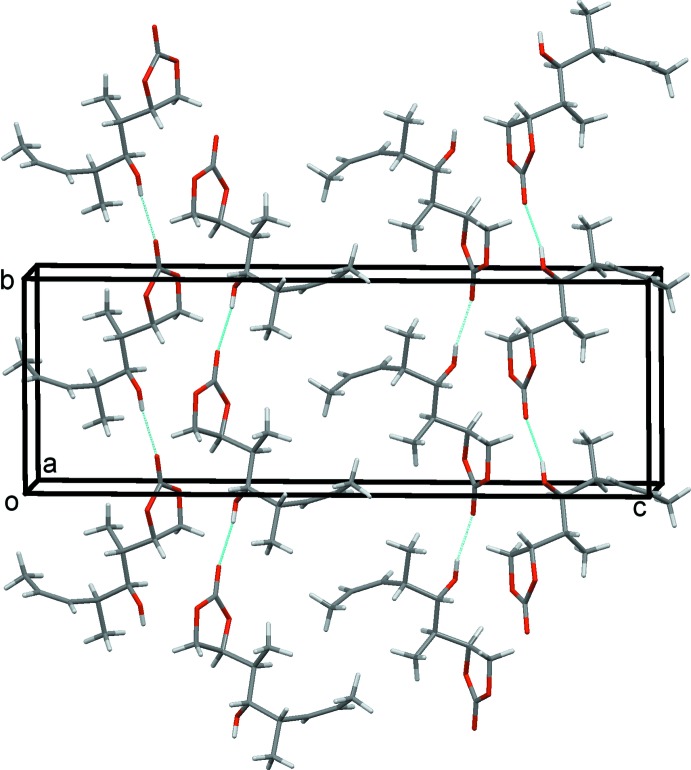
A view along the *a* axis of crystal packing of the title compound, with hydrogen bonds shown as dashed lines (see Table 1 ▸).

The conformational distance between the hydroxyl group and the carbonyl moiety does not allow intra­molecular hydrogen-bond formation, therefore, hydrogen bonding is observed through inter­molecular inter­actions alone (Table 1 ▸). In the crystal, neighbouring mol­ecules are linked by the O4—H4⋯O1^i^ hydrogen bond, forming chains along [010]; see Fig. 2 ▸ and Table 1 ▸.

## Database survey   

A search of the Cambridge Structural Database (Version 5.38, updated May 2017; Groom *et al.*, 2016 ▸) revealed no related compounds with the 3-hy­droxy-2-methyl-1,2-carbonate substructure. However, a search for the 2,4-di­methyl­hex-5-en-3-ol fragment revealed more than 120 hits. Many of these involve reactants for the synthesis of natural products, such as superotolide A (Yakelis & Roush, 2003 ▸) and erythronolides A and B (Lynch *et al.*, 1989*a* ▸; 1989*b* ▸).

## Synthesis and crystallization   

**Figure 3:**
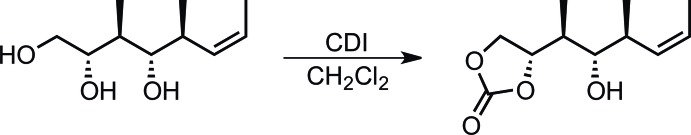
Reaction scheme

The synthesis of the title compound, illustrated in Fig. 3 ▸, was performed through the selective protection of the 1,2-diol of (+)-(2*S*,3*S*,4*S*,5*S*,*Z*)-3,5-di­methyl­oct-6-ene-1,2,4-triol with a carbonate using *N*,*N′*-carbonyl­diimidazole (CDI) in CH_2_Cl_2_ as solvent, favouring formation of the 1,2-carbonate over the 1,3-carbonate. This reaction afforded the optically active *anti,anti,anti*-polypropionate unit with the correct absolute configuration. To a dry round-bottom flask containing the 1,2-diol of (+)-(2*S*,3*S*,4*S*,5*S*,*Z*)-3,5-di­methyl­oct-6-ene-1,2,4-triol (0.04 g, 0.212 mmol) in dry CH_2_Cl_2_ (1.07 ml, 0.2 *M*) was added *N,N′*-carbonyl­diimidazole (0.048 g, 0.30 mmol). The reaction mixture was stirred at 298 K for 2.5 h, then saturated aqueous NaCl was added. The resulting mixture was then extracted with ethyl acetate (three times). The combined organic layer was dried over MgSO_4_ and concentrated at reduced pressure. The crude product was purified by flash chromatography (2:1, ethyl acetate:hexa­ne) to yield 0.027 g (62%) of the pure title carbonate product as a white solid (m.p. 360–363 K). Block-like clear crystals suitable for X-ray diffraction, were obtained by slow diffusion of a 1:1 (*v*:*v*) ethyl acetate:hexa­nes solution of the title compound at room temperature over a period of two days. NMR analyses were performed on a Bruker AV-500 spectrometer using Chloro­form-*d* as solvent (CDCl_3_). The solvent signal at 7.26 and 77.00 ppm were used as inter­nal standards for proton and carbon respectively. ^1^H NMR (500 MHz, CDCl_3_) δ 5.69 (*dq*, *J* = 10.9, 6.8 Hz, 1H), 5.23 (*ddt*, *J* = 11.2, 9.8, 1.8 Hz, 1H), 4.99 (*td*, *J* = 8.2, 5.0 Hz, 1H), 4.44 (*t*, *J* = 8.6 Hz, 1H), 4.37 (*t*, *J* = 8.6 Hz, 1H), 3.26 (*dd*, *J* = 7.5, 4.2 Hz, 1H), 2.72 (*ddq*, *J* = 6.9, 6.7, 3.1 Hz, 1H), 2.29 (*ddq*, *J* = 6.6, 4.5, 2.4 Hz, 1H), 2.00 (*s*, 1H, -OH), 1.65 (*dd*, *J* = 6.8, 1.9 Hz, 3H), 1.05 (*d*, *J* = 6.9 Hz, 3H), 1.00 (d, *J* = 6.6 Hz, 3H). ^13^C NMR (125 MHz, CDCl_3_) δ 155.3, 131.3, 127.4, 77.5, 77.3, 66.9, 36.8, 35.3, 17.1, 13.3, 11.7. [α]^20^_D_ = −2.0 (*c* = 1.0, CHCl_3_). Analysis calculated for C_11_H_18_O_4_: C, 61.66, H, 8.47%. Found: C, 61.74, H, 8.44%. IR data: C=O: 1761.32 cm^−1^, C—O: 1061.01 cm^−1^.

## Refinement   

Crystal data, data collection and structure refinement details are summarized in Table 2 ▸. H atoms were included in geometrically calculated positions and refined as riding: O—H = 0.82 Å, C—H = 0.93–0.98 Å with *U*_iso_(H) = 1.5*U*_eq_(O-hydroxyl and C-meth­yl) and 1.2*U*_eq_(C) for other H atoms.

## Supplementary Material

Crystal structure: contains datablock(s) Global, I. DOI: 10.1107/S2056989017009318/su5376sup1.cif

Structure factors: contains datablock(s) I. DOI: 10.1107/S2056989017009318/su5376Isup2.hkl

Supporting information file. DOI: 10.1107/S2056989017009318/su5376Isup3.cml

CCDC reference: 1548935

Additional supporting information:  crystallographic information; 3D view; checkCIF report

## Figures and Tables

**Table 1:** Hydrogen-bond geometry (Å, °)

*D*—H⋯*A*	*D*—H	H⋯*A*	*D*⋯*A*	*D*—H⋯*A*
O4—H4⋯O1^i^	0.82	2.05	2.811 (2)	155

Symmetry code: (i) 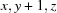.

**Table 2:** Experimental details

Crystal data	
Chemical formula	C_11_H_18_O_4_
*M*_r_	214.25
Crystal system, space group	Orthorhombic, *P*2_1_2_1_2_1_
Temperature (K)	100
*a*, *b*, *c* (Å)	5.0968 (1), 8.8153 (1), 25.6052 (3)
*V* (Å^3^)	1150.44 (3)
*Z*	4
Radiation type	Cu *K*α
μ (mm^−1^)	0.77
Crystal size (mm)	0.23 × 0.13 × 0.06

Data collection	
Diffractometer	Rigaku OD SuperNova, single source at offset/far, HyPix3000
Absorption correction	Gaussian (*CrysAlis PRO*; Rigaku OD, 2016 ▸)
*T*_min_, *T*_max_	0.739, 1.000
No. of measured, independent and observed [*I* > 2σ(*I*)] reflections	17757, 2131, 2081
*R*_int_	0.030
(sin θ/λ)_max_ (Å^−1^)	0.606

Refinement	
*R*[*F* ^2^ > 2σ(*F* ^2^)], *wR*(*F* ^2^), *S*	0.032, 0.094, 1.26
No. of reflections	2131
No. of parameters	141
H-atom treatment	H-atom parameters constrained
Δρ_max_, Δρ_min_ (e Å^−3^)	0.23, −0.17
Absolute structure	Flack *x* determined using 812 quotients [(*I* ^+^)−(*I* ^−^)]/[(*I* ^+^)+(*I* ^−^)] (Parsons *et al.*, 2013 ▸)
Absolute structure parameter	0.05 (6)

Computer programs: *CrysAlis PRO* (Rigaku OD, 2016 ▸), *SHELXT* (Sheldrick, 2015*a* ▸), *SHELXL2016* (Sheldrick, 2015*b* ▸), *Mercury* (Macrae *et al.*, 2008 ▸) and *OLEX2* (Dolomanov *et al.*, 2009 ▸).

## supplementary crystallographic information

### Crystal data

**Table:**

C_11_H_18_O_4_	*D*_x_ = 1.237 Mg m^−^^3^
*M**_r_* = 214.25	Cu *K*α radiation, λ = 1.54184 Å
Orthorhombic, *P*2_1_2_1_2_1_	Cell parameters from 12810 reflections
*a* = 5.0968 (1) Å	θ = 3.5–68.8°
*b* = 8.8153 (1) Å	µ = 0.77 mm^−^^1^
*c* = 25.6052 (3) Å	*T* = 100 K
*V* = 1150.44 (3) Å^3^	Block, colourless
*Z* = 4	0.23 × 0.13 × 0.06 mm
*F*(000) = 464	

### Data collection

**Table:**

Rigaku OD SuperNova, Single source at offset/far, HyPix3000 diffractometer	2131 independent reflections
Radiation source: micro-focus sealed X-ray tube, SuperNova (Cu) X-ray Source	2081 reflections with *I* > 2σ(*I*)
Mirror monochromator	*R*_int_ = 0.030
ω scans	θ_max_ = 69.0°, θ_min_ = 3.5°
Absorption correction: gaussian (CrysAlis PRO; Rigaku OD, 2016)	*h* = −6→6
*T*_min_ = 0.739, *T*_max_ = 1.000	*k* = −10→10
17757 measured reflections	*l* = −30→31

### Refinement

**Table:**

Refinement on *F*^2^	H-atom parameters constrained
Least-squares matrix: full	*w* = 1/[σ^2^(*F*_o_^2^) + (0.0367*P*)^2^ + 0.4414*P*] where *P* = (*F*_o_^2^ + 2*F*_c_^2^)/3
*R*[*F*^2^ > 2σ(*F*^2^)] = 0.032	(Δ/σ)_max_ < 0.001
*wR*(*F*^2^) = 0.094	Δρ_max_ = 0.23 e Å^−^^3^
*S* = 1.26	Δρ_min_ = −0.17 e Å^−^^3^
2131 reflections	Extinction correction: (SHELXL2016; Sheldrick, 2015b), Fc^*^=kFc[1+0.001xFc^2^λ^3^/sin(2θ)]^-1/4^
141 parameters	Extinction coefficient: 0.0032 (6)
0 restraints	Absolute structure: Flack *x* determined using 812 quotients [(*I*^+^)-(*I*^-^)]/[(*I*^+^)+(*I*^-^)] (Parsons *et al.*, 2013)
Primary atom site location: dual	Absolute structure parameter: 0.05 (6)
Hydrogen site location: inferred from neighbouring sites	

### Special details

**Table:**

Geometry. All esds (except the esd in the dihedral angle between two l.s. planes) are estimated using the full covariance matrix. The cell esds are taken into account individually in the estimation of esds in distances, angles and torsion angles; correlations between esds in cell parameters are only used when they are defined by crystal symmetry. An approximate (isotropic) treatment of cell esds is used for estimating esds involving l.s. planes.

### Fractional atomic coordinates and isotropic or equivalent isotropic displacement parameters (Å^2^)

**Table:**

	*x*	*y*	*z*	*U*_iso_*/*U*_eq_	
O1	0.9031 (4)	−0.13807 (18)	0.69895 (7)	0.0267 (4)	
O2	0.6220 (3)	0.03761 (19)	0.72821 (6)	0.0234 (4)	
O3	0.9752 (3)	0.10820 (18)	0.68416 (6)	0.0214 (4)	
O4	0.8749 (4)	0.55481 (18)	0.66999 (6)	0.0241 (4)	
H4	0.838292	0.644582	0.674153	0.036*	
C1	0.8397 (5)	−0.0079 (3)	0.70337 (9)	0.0196 (5)	
C2	0.6171 (5)	0.2019 (3)	0.73034 (9)	0.0208 (5)	
H2A	0.653581	0.237855	0.765407	0.025*	
H2B	0.447987	0.240857	0.719285	0.025*	
C3	0.8339 (5)	0.2503 (2)	0.69242 (9)	0.0182 (5)	
H3	0.949859	0.323486	0.709691	0.022*	
C4	0.7482 (5)	0.3135 (2)	0.63967 (8)	0.0164 (5)	
H4A	0.904016	0.316110	0.617275	0.020*	
C5	0.6564 (5)	0.4780 (2)	0.64684 (9)	0.0179 (5)	
H5	0.509516	0.479338	0.671547	0.021*	
C6	0.5674 (5)	0.5528 (3)	0.59550 (8)	0.0188 (5)	
H6	0.418322	0.494773	0.581917	0.023*	
C7	0.7832 (5)	0.5488 (3)	0.55492 (9)	0.0224 (5)	
H7	0.947944	0.581376	0.565794	0.027*	
C8	0.7641 (5)	0.5043 (3)	0.50573 (9)	0.0266 (5)	
H8	0.916088	0.510563	0.485756	0.032*	
C9	0.5243 (6)	0.4446 (4)	0.47858 (10)	0.0394 (7)	
H9A	0.492138	0.503209	0.447625	0.059*	
H9B	0.375977	0.452228	0.501530	0.059*	
H9C	0.551540	0.340378	0.469213	0.059*	
C10	0.5456 (5)	0.2129 (3)	0.61247 (9)	0.0208 (5)	
H10A	0.379257	0.222893	0.629735	0.031*	
H10B	0.601834	0.109062	0.613932	0.031*	
H10C	0.528349	0.243598	0.576661	0.031*	
C11	0.4749 (6)	0.7165 (3)	0.60482 (10)	0.0268 (6)	
H11A	0.343748	0.717439	0.631719	0.040*	
H11B	0.401916	0.756573	0.573128	0.040*	
H11C	0.621298	0.777709	0.615420	0.040*	

### Atomic displacement parameters (Å^2^)

**Table:**

	*U*^11^	*U*^22^	*U*^33^	*U*^12^	*U*^13^	*U*^23^
O1	0.0337 (10)	0.0144 (8)	0.0319 (9)	0.0021 (7)	−0.0088 (8)	−0.0007 (7)
O2	0.0230 (8)	0.0183 (8)	0.0289 (8)	−0.0017 (7)	0.0033 (7)	0.0048 (7)
O3	0.0198 (8)	0.0151 (7)	0.0292 (8)	0.0029 (7)	0.0022 (7)	0.0037 (6)
O4	0.0278 (9)	0.0128 (7)	0.0316 (9)	0.0000 (7)	−0.0107 (7)	−0.0023 (7)
C1	0.0218 (11)	0.0181 (11)	0.0189 (10)	−0.0010 (10)	−0.0052 (9)	0.0004 (9)
C2	0.0244 (12)	0.0166 (11)	0.0215 (11)	0.0024 (11)	0.0028 (10)	0.0003 (9)
C3	0.0180 (11)	0.0134 (10)	0.0231 (11)	0.0004 (9)	−0.0006 (9)	−0.0017 (8)
C4	0.0159 (10)	0.0128 (10)	0.0205 (10)	0.0007 (9)	0.0012 (9)	−0.0006 (8)
C5	0.0175 (11)	0.0146 (10)	0.0214 (11)	−0.0010 (9)	−0.0012 (9)	−0.0014 (9)
C6	0.0158 (11)	0.0181 (11)	0.0224 (11)	−0.0002 (9)	−0.0019 (9)	0.0013 (9)
C7	0.0164 (11)	0.0230 (11)	0.0277 (11)	0.0002 (10)	−0.0008 (9)	0.0054 (10)
C8	0.0217 (12)	0.0331 (13)	0.0251 (11)	0.0042 (11)	0.0026 (10)	0.0053 (10)
C9	0.0310 (15)	0.0616 (19)	0.0258 (13)	−0.0003 (15)	−0.0020 (11)	−0.0064 (13)
C10	0.0218 (12)	0.0182 (11)	0.0224 (11)	−0.0015 (10)	−0.0008 (9)	−0.0013 (9)
C11	0.0314 (14)	0.0192 (12)	0.0299 (12)	0.0065 (11)	−0.0050 (11)	0.0029 (10)

### Geometric parameters (Å, º)

**Table:**

O1—C1	1.198 (3)	C6—H6	0.9800
O2—C1	1.340 (3)	C6—C7	1.513 (3)
O2—C2	1.450 (3)	C6—C11	1.536 (3)
O3—C1	1.329 (3)	C7—H7	0.9300
O3—C3	1.461 (3)	C7—C8	1.323 (3)
O4—H4	0.8200	C8—H8	0.9300
O4—C5	1.432 (3)	C8—C9	1.501 (4)
C2—H2A	0.9700	C9—H9A	0.9600
C2—H2B	0.9700	C9—H9B	0.9600
C2—C3	1.532 (3)	C9—H9C	0.9600
C3—H3	0.9800	C10—H10A	0.9600
C3—C4	1.525 (3)	C10—H10B	0.9600
C4—H4A	0.9800	C10—H10C	0.9600
C4—C5	1.535 (3)	C11—H11A	0.9600
C4—C10	1.529 (3)	C11—H11B	0.9600
C5—H5	0.9800	C11—H11C	0.9600
C5—C6	1.539 (3)		
			
C1—O2—C2	109.32 (19)	C5—C6—H6	107.9
C1—O3—C3	110.52 (17)	C7—C6—C5	111.25 (19)
C5—O4—H4	109.5	C7—C6—H6	107.9
O1—C1—O2	123.7 (2)	C7—C6—C11	110.58 (19)
O1—C1—O3	124.2 (2)	C11—C6—C5	111.11 (18)
O3—C1—O2	112.07 (19)	C11—C6—H6	107.9
O2—C2—H2A	111.0	C6—C7—H7	116.3
O2—C2—H2B	111.0	C8—C7—C6	127.4 (2)
O2—C2—C3	104.01 (18)	C8—C7—H7	116.3
H2A—C2—H2B	109.0	C7—C8—H8	116.4
C3—C2—H2A	111.0	C7—C8—C9	127.2 (2)
C3—C2—H2B	111.0	C9—C8—H8	116.4
O3—C3—C2	102.04 (17)	C8—C9—H9A	109.5
O3—C3—H3	109.4	C8—C9—H9B	109.5
O3—C3—C4	109.03 (18)	C8—C9—H9C	109.5
C2—C3—H3	109.4	H9A—C9—H9B	109.5
C4—C3—C2	117.2 (2)	H9A—C9—H9C	109.5
C4—C3—H3	109.4	H9B—C9—H9C	109.5
C3—C4—H4A	107.1	C4—C10—H10A	109.5
C3—C4—C5	109.04 (18)	C4—C10—H10B	109.5
C3—C4—C10	112.68 (18)	C4—C10—H10C	109.5
C5—C4—H4A	107.1	H10A—C10—H10B	109.5
C10—C4—H4A	107.1	H10A—C10—H10C	109.5
C10—C4—C5	113.36 (19)	H10B—C10—H10C	109.5
O4—C5—C4	105.02 (18)	C6—C11—H11A	109.5
O4—C5—H5	108.7	C6—C11—H11B	109.5
O4—C5—C6	112.33 (18)	C6—C11—H11C	109.5
C4—C5—H5	108.7	H11A—C11—H11B	109.5
C4—C5—C6	113.11 (18)	H11A—C11—H11C	109.5
C6—C5—H5	108.7	H11B—C11—H11C	109.5
			
O2—C2—C3—O3	−13.8 (2)	C2—C3—C4—C10	−49.2 (3)
O2—C2—C3—C4	105.2 (2)	C3—O3—C1—O1	175.2 (2)
O3—C3—C4—C5	−167.31 (18)	C3—O3—C1—O2	−4.5 (2)
O3—C3—C4—C10	65.9 (2)	C3—C4—C5—O4	56.7 (2)
O4—C5—C6—C7	61.8 (2)	C3—C4—C5—C6	179.52 (19)
O4—C5—C6—C11	−61.9 (3)	C4—C5—C6—C7	−56.9 (3)
C1—O2—C2—C3	12.2 (2)	C4—C5—C6—C11	179.4 (2)
C1—O3—C3—C2	11.6 (2)	C5—C6—C7—C8	131.2 (3)
C1—O3—C3—C4	−113.0 (2)	C6—C7—C8—C9	−1.0 (4)
C2—O2—C1—O1	174.9 (2)	C10—C4—C5—O4	−176.93 (18)
C2—O2—C1—O3	−5.3 (2)	C10—C4—C5—C6	−54.1 (3)
C2—C3—C4—C5	77.5 (2)	C11—C6—C7—C8	−104.8 (3)

### Hydrogen-bond geometry (Å, º)

**Table:**

*D*—H···*A*	*D*—H	H···*A*	*D*···*A*	*D*—H···*A*
O4—H4···O1^i^	0.82	2.05	2.811 (2)	155

Symmetry code: (i) *x*, *y*+1, *z*.
